# Qualitative methods are epidemiologic methods: Revisiting the epidemiologist's toolbox

**DOI:** 10.1093/aje/kwaf083

**Published:** 2025-04-29

**Authors:** Elisabeth A Stelson, Roxanne Dupuis

**Affiliations:** Washington University in St. Louis, School of Public Health, St. Louis, MO, United States; Department of Epidemiology, T.H. Chan School of Public Health, Harvard University, Boston, MA, United States; Department of Population Health, NYU Grossman School of Medicine, New York, NY, United States

**Keywords:** qualitative research, epidemiology, causal inference, public health, methodology

## Abstract

Qualitative research methods are frequently described as “compatible” with quantitative epidemiologic methods. Instead of simply “compatible,” we argue that qualitative methods *are* epidemiologic methods. Especially in social epidemiology, which embraces the relationships between psychosocial, historical, contextual, and intersectional factors and health, qualitative research methods have the potential to provide a more complete picture of the distribution of health and disease within a population and contexts contributing to population health. To this end, this paper compares qualitative research and epidemiologic research definitions, outlines epidemiologic uses of qualitative data, and addresses common concerns and misconceptions about qualitative research. We emphasize the shared characteristics and champion the use of shared standards across qualitative and quantitative approaches in epidemiology.

This article is part of a Special Collection on Methods in Social Epidemiology.

## Introduction

Qualitative methods are frequently described as “compatible” with quantitative epidemiologic methods. Instead of simply “compatible,” we argue that qualitative methods *are* epidemiologic methods. To describe them as “compatible” relegates these methods to the sidelines and minimizes their importance to identifying the *how*, *what*, and *why* that allows epidemiologic research to be actionable, consequential, and translate into good public health practice.^1-3^ While qualitative methods may increasingly be used in epidemiologic research focusing on social phenomena (ie, social epidemiology),^4^ they are also valuable for clinical and field epidemiology.^5^^,^^6^

Before returning to this argument, we thought it helpful to review definitions of *qualitative methods*, *epidemiology*, *social epidemiology,* and *empirical research*. In their foundational text, Straus and Corbin define *qualitative research* as “a nonmathematical process of interpretation, carried out for the purpose of discovering concepts and relationships in raw data, and then organizing these into a theoretical explanatory scheme.”^7^ But just because qualitative data may be “nonmathematical” does not mean that it is not systematic and scientific.^8^^,^^9^ Qualitative methods are a family of different approaches that a researcher can choose from based on a study’s research question (Sound familiar? This is what guides the selection of a quantitative approach, too). While this paper is not designed to be a primer on these methods, Table 1 offers a brief description of common qualitative methods. Just as quantitative methods can be used in cross-sectional or longitudinal studies, or in samples matched or stratified based on some attribute, so too, can interviews, focus groups, and other qualitative methods.

**Table 1:** Brief descriptions of common qualitative data collection methods that can be used in epidemiologic research.

**Qualitative methods**	**Description**
Document analysis	Analyzing existing printed and electronic documents for deeper understanding and patterns.
Focus group	Guided conversation with a group of research participants (usually 6-12 people) facilitated by a researcher.
Free listing	Technique used in interviews, focus groups, or surveys in which participants are asked to list all words they can think of related to a topic.
Interview	One-on-one guided conversations with research participant. Examples include depth, life course, key informant, and cognitive.
Nominal group technique	Structured method for group brainstorming and topic prioritization that encourages contributions from each participant while minimizing power dynamics.
Participant observation	Researcher immerses themselves in a particular setting and records observations pertaining to behaviors and interactions between people and with their environments.
Photo elicitation	Type of interview guided by questions pertaining to photos viewed by both the participant and the researcher.
Photo voice	Type of interview guided by questions pertaining to photos taken or chosen by the research participant. Participants are often also involved in interpreting and disseminating the analysis.
Written response	Text written by participant, often in response to an open-ended survey question.

The Centers for Disease Control and Prevention (CDC) define *epidemiology* as “the study (scientific, systematic, and data-driven) of the distribution (frequency, pattern) and determinants (causes, risk factors) of health-related states and events (not just diseases) in specified populations (neighborhood, school, city, state, country, global).”^10^ Epidemiology textbooks offer similar definitions.^11-13^ While the emphasis on “frequency” in this (and other) definitions of epidemiology may lend itself more easily to quantitative methods, it is only one component of the definition. Indeed, identifying “patterns” is the bread-and-butter of qualitative methods, particularly qualitative content analysis.^14^  *Social epidemiology*, for its part, focuses on the ways that structural, institutional, and relational factors shape the health of populations.^15^ Social epidemiology is particularly suited to the use of both qualitative and quantitative data that, as Schwab and Syme write, “reflect the ecological reality of life in [the] population, as people experience it” (p. 2050).^16^

There are similarities across the definitions of qualitative research and epidemiologic research. Both definitions emphasize the systematic and scientific analysis of data used to explain what is happening in our world and the people in it. Both are *empirical*, defined by the Oxford English Dictionary as “based on, concerned with, or verifiable by observation or experience rather than theory or pure logic.”^17^ Importantly, there is nothing about the definition of *epidemiology* that is inconsistent with the uses and application of qualitative methods or that specifies the use of quantitative methods.

Numerous researchers have argued over the past two decades that qualitative methods are *complementary* to epidemiology and should be used alongside epidemiologic methods to enhance understanding of health phenomena.^8^^,^^13^^,^^18-20^ Such statements are usually accompanied by descriptions, at least stereotypically, of two separate—and sometimes incompatible—research cultures: quantitative researchers who adopt a positivistic perspective of the world and qualitative researchers who interpret the world through a more constructionist lens.^21-26^ But with growth and advances in social epidemiology, which embraces relationships between psychosocial, historical, contextual, and intersectional factors and health, the strict positivist worldview among epidemiologists is being challenged.^27-31^ We contend that relegation of qualitative methods to the “complementary” role is more a relic of research cultures than something inherent in the methods themselves.

The consequence of conceptualizing qualitative methods as “compatible” but outside the epidemiologist's toolbox is that qualitative methods are conceived of as expendable and nonessential. It also places qualitative methods either outside of the traditional hierarchy of evidence or denigrates it as one of the weaker designs. As a result, the professional recognition and reward for such work is diminished for epidemiologists, thereby disincentivizing its use and leading to its omission in many epidemiology training programs.^32^^,^^33^ This is not just an academic debate or professional conundrum, but rather an issue that has very real implications for translating epidemiology into public health practice.^34^ As Adkins-Jackson et al. cogently write, qualitative data can “corroborate, dispute, or delineate the practices and policies that create, sustain, and reinforce health inequities […] connect[ing] structural factors (eg, policies) to individual outcomes” (p. 542).^25^ Without using qualitative methods, epidemiologists risk overlooking key perspectives of people most affected and knowledgeable of an issue—knowledge which can ensure we ask the right questions, measure appropriately, and interpret results accurately and responsibly to improve public health programs, polices, and practices.^25^^,^^35^^,^^36^

Qualitative formative research and process evaluation have often been used in intervention research—a suitable application of these methods. As we and others argue, qualitative methods are also essential tools for identifying causal mechanisms and pathways in observational settings and for intervention dissemination.^4^^,^^22^^,^^24^^,^^37^^,^^38^ Without the embrace of these methods as our own, epidemiologists'—including social and clinical epidemiologists—impact on public health is hampered. As Bannister-Tyrrell and Meigari write: “As long as qualitative research is constrained to being part of interdisciplinary endeavors only, the full potential of qualitative research in epidemiology will not be realized. This may only change when we move from asking, ‘are qualitative methodologies applicable to answer epidemiological questions?’ to ‘how and what types of qualitative research are applicable to answer a specific epidemiological question?’” (p. 34).^22^

Instead of valuing quantitative and qualitative methods differently, we emphasize their shared characteristics and champion the use of shared standards across methodological approaches to ensure their application “leads to meaningful conceptualization and measurement, interpretable causal inferences, and a better understanding” (p. xv) of epidemiologic phenomena as Brady and Collier suggest.^38^ To this end, this paper starts by outlining some of the epidemiologic uses of qualitative data. We then address misconceptions of qualitative data within the field of epidemiology to alleviate concerns epidemiologists may have in adopting these methods.

## Epidemiological uses of qualitative methods

### Use 1: hypothesis generation

Qualitative data are (and have been) useful in making observations about the world and generating hypotheses about the distribution and determinants of health and disease. Qualitative methods can help scientists identify the problem, ascertain what requires measurement and why, and, if future studies will be quantitative in nature, select the appropriate variables to include. Take, for example, Scoglio et al.'s qualitative analysis of open-ended *Nurses’ Health Study* survey responses of healthcare workers during the COVID-19 pandemic. In this study, role conflict surfaced as a defining occupational theme among participants with high moral injury scores. This qualitative finding was then quantitatively operationalized and tested, finding that high role conflict significantly contributed to moral injury symptoms.^39^

### Use 2: strengthen study design

Qualitative methods are also useful throughout the study design and data collection process. First, these methods can be used to vet data collection instruments, including surveys or scales. To this end, cognitive interviews are a useful qualitative approach to test instruments for clarity and comprehension by potential study participants, thereby ensuring that the data collected is indeed what was intended by the research team.^40^ For example, Harnois's cognitive interviews of racially diverse adults completing the Everyday Discrimination Scale, an instrument frequently used in social epidemiology,^41^ illustrated how individuals interpret the items in vastly different ways, likely depending on their experience of their race and gender. Harnois concludes that the instrument may not consistently measure everyday discrimination as many quantitative social epidemiologic studies have assumed.^40^

Second, qualitative methods can be used to help reach the study population. Specifically, key informant interviews can help researchers understand how to recruit “hard-to-reach” populations to ensure they are represented in samples.^42^ These interviews can also snowball into connections with members of those populations and create buy-in for a specific study. Third, obtaining participant perspectives prior to study implementation could also identify potential issues with selection bias or social desirability bias ahead of time, allowing the research team to update their recruitment methods and/or data collection tools.^43^

### Use 3: strengthen intervention design, implementation, and dissemination

Qualitative methods can strengthen the design and implementation of interventions that epidemiologists evaluate in trials or in the field.^6^^,^^44^ Qualitative methods can illicit from potential study participants and community members nuanced descriptions of the context within which they are situated, feasibility and acceptability of an intervention, and how this might affect intervention participation.^45^^,^^46^ More specifically, qualitative interviews might highlight participants’ competing priorities and how these may interfere with adherence to a specific intervention.^47^ Focus group discussions may identify common barriers and facilitators to specific behavior change.^48^ Putting this into practice, the CDC Field Epidemiology Manual explicitly suggests that field epidemiologists have a firm grasp of qualitative methods so they can understand the sociopolitical context of urgent public health problems and respond, adapt, and implement effective interventions.^6^^,^^44^

Qualitative methods can also provide meaningful insight into how to disseminate and implement an intervention if proven effective. Interventions tested in controlled environments are often less effective when implemented in communities.^49^ This can be due to access barriers or components of study design influencing participant behavior, among other factors. The use of interviews, focus groups, and content analysis of study materials can help elucidate potential challenges with future dissemination and implementation.^34^ For example, a qualitative study of children and parents participating in a comparative effectiveness trial testing two mental health interventions identified how components of the study design—not the intended intervention—provided additional social support, thereby making it different from the disseminated version of the intervention.^34^

### Use 4: descriptive inference

Descriptive inference may be considered a more typical use of qualitative data. While descriptive quantitative epidemiologic studies may report summary statistics to describe the distribution of health and disease in the population, qualitative data that collects in-depth personal experiences described in participants' own words can provide nuanced descriptions of complex phenomena, which may involve interdependent effects across time and space.^3^^,^^50^ These rich descriptions can provide insights into the root causes of racial, ethnic, gender, disability, etc. health inequities and intersections thereof, critical for many social epidemiologic studies.^25^ For example, in an in-depth interview study of predominately African American mothers released from jail, participants reported how they experienced few barriers to healthcare for themselves or their children upon reentry, but they described a diversity of barriers to housing, employment, and other social determinants of health that they reported affected their health and wellbeing post-release.^51^

### Use 5: causal inference and mechanistic understanding of causal processes

Causality is a theoretical concept rather than a methodological one.^9^ There are instances when it may not be feasible to do causal inference using qualitative data or where quantitative data may be more suitable to answering a research question. In such instances, qualitative data can be used to interpret the values, their significance (clinically rather than statistically), and the processes that led to them. This is an extension of the mechanistic understanding of causal processes.^52^^,^^53^ In the Learning Together whole-school intervention, for example, Warren et al. used interviews and focus groups to elicit the mechanisms through which an intervention consisting of restorative practice training for staff and a social and emotional learning curriculum for students reduced experiences of bullying.^54^

Qualitative data can also be used to assess whether quantitative results and inferences about those results are “true” or whether they are an artifact of study design or proxy measures.^4^^,^^22^ We can do this by eliciting feedback from study participants through various qualitative methods, including embedding open-ended responses in surveys.^55^^,^^56^ Member checking, a process whereby researchers share study results with participants or community members and qualitatively elicit feedback on accuracy and validity of interpretations via interviews, focus groups, or other techniques, can also be an effective tool for understanding causal processes and alternative explanations.^57^ Grounding discussion of quantitative results in existing qualitative literature can likewise yield important insight. For greater elaboration on using qualitative methods for causal inference we refer readers to Houghton and Paniagua-Avila^4^ and Bannister-Tyrrell and Meiqari.^22^

## Debunking common concerns and misconceptions

While readers may acknowledge the methodological contributions of qualitative methods described above, they may still fixate on several common concerns and misconceptions that make qualitative methods appear less rigorous. In this section, we outline the most common concerns, discuss how these concerns may also be present in quantitative research, and identify ways to minimize their effect.

### Concern 1: analysis is not systematic

A common misconception of qualitative research is that data collection and analysis is not systematic. To this we say: it is systematic … if data collection and analysis is done well. With person-facing data collection (like interviews and focus groups), the researcher follows an interview guide to identify what topics need to be discussed and specific areas of follow up.^20^ Depending on the method, these questions may be asked in different orders to maximize participant comfort and improve data quality or potentially even skipped if the participant answers the question without prompting.^58^ However, the researcher still utilizes the guide as a systematic data collection tool. Any changes and decision in data collection or the study as a whole are documented in an audit trail.^59^

Analysis of qualitative data is likewise systematic. There are multiple approaches to systematically analyze qualitative data after it has been collected. In one such approach for text data (eg, interview transcripts), the research team develops a codebook with specific inclusion/exclusion criteria to determine how the text should be categorized (ie, coded). These codes and their definition may be based on topics that organically surface from review of the data, a priori constructs pertaining to the research question, theoretical constructs guiding the research, or any combination thereof. Detail and number of codes may differ based on analytic approach (template coding, framework analysis, etc.).^60-62^ The process for identifying codes and deciding on the number of codes to include in a codebook is analogous to deciding what variables are important to include in quantitative analyses. The codebook is then consistently applied to all the data. There is the possibility that data could be miscoded, misinterpreted, or interpreted differently, akin to issues of misclassification error with quantitative data analysis. To guard against this (and to also identify areas of meaningful difference in interpretation), two researchers often independently code the data.^63^ Advances in qualitative analysis software allow for coding comparison (eg, calculating interrater reliability) throughout the coding process to ensure consistency.^64^

### Concern 2: researcher hears what they want to hear

Like with concerns about the systematic nature of qualitative research, this concern is only a concern if qualitative research is done poorly. As humans, we all have our perspectives and assumptions, and this can shape how and what data are collected, as well as how we interpret that data.^65^^,^^66^ This is true for both qualitative and quantitative research. However, unlike quantitative research, qualitative research explicitly creates space to discuss researchers' subjectivity, positionality, and assumptions and make this clear for readers.^67^ We suggest that this is a practice that quantitative research teams may benefit from borrowing to improve and reflect upon their choice of variables and interpretation of findings.

Qualitative research is often done in teams, which allows researchers to discuss approaches to data collection and interpretation of data with one another.^62^^,^^67^ Iterative reflective discussion throughout all phases of the research process is a key safeguard against “hearing what you want to hear.” Steps described above to develop a codebook with clear inclusion/exclusion coding criteria and to calculate interrater reliability are also excellent ways to protect against this form of confirmation bias.

Perhaps, underpinning this concern is a mistaken belief that just about anybody can conduct this type of research, since it is “just talking to people.” Just as a researcher requires specific statistical training to employ marginal structural models, so too, do researchers require training specific to the type of qualitative method, such as photovoice or nominal group technique, they hope to use.^35^ Recently, there has been a mild backlash against including qualitative methods within quantitative research, often because this mixed-method analysis is not done in a thoughtful way.^35^^,^^68^ Such concerns are justified, but overlook the root cause of poorly-conducted mixed-method research: we do not typically train epidemiologists in qualitative methods.^32^^,^^33^

### Concern 3: small sample size

The small sample sizes typical of qualitative analysis sometimes shock quantitative researchers accustomed to large datasets. However, with qualitative analysis, the sample size is determined through saturation rather than statistical power. Saturation is defined as the point at which the inclusion of new data produces little to no new information, thereby diminishing the utility of including more participants in the study.^69^ Whereas statistical power can be calculated a priori based upon the number of variables, distribution of data, and effect size, saturation is assessed iteratively throughout the data collection and analysis process based on the amount of detail collected, specificity of the research question, and heterogeneity of participants. Whereas increasing sample size will always improve precision in quantitative analysis,^70^ quality of qualitative analysis may not be improved after a certain sample size is reached and will only increase participant (and researcher) burden.^71^^,^^72^ In sum, size does matter, but bigger is not always better. That said, participant characteristics essential to the research questions can guide preliminary recruitment planning. For example, for an interview-based study in which geography or job type is important to the research question, the research team may aim to recruit 10 to 12 participants from each geographic or job strata and then assess if additional data collection is necessary based upon whether new information arose with each subsequent interview for each of these strata.^73^

### Concern 4: participants are not randomly sampled

Quantitative researchers are often hesitant to rely on the results of qualitative studies because participants are not randomly sampled, potentially introducing selection bias. Considering who is and is not in a study *and* why is critical to identifying application and limitations of research findings. As is the case with observational quantitative studies with hard-to-reach populations or undelimitated sampling frames, sometimes it is not possible to randomly sample participants. Moreover, random selection does not inherently mean the sample will approximate the true population due to nonresponse and attrition.^74^ Additionally, there are instances in both quantitative and qualitative research where a random sample is not the goal, such as when one is actively trying to disprove cases, in which case a random sample may weaken the study design.^75^

That said, the belief that qualitative research is inherently non-random is inaccurate. Just like with other quantitative designs, participants can be randomly selected to participate in a qualitative study^76^ or may have been randomly assigned to an intervention in a mixed method randomized study.^48^^,^^77^ Ultimately, the utility of random sampling in qualitative research depends on the research question.

### Concern 5: data are self-reported

Many quantitative researchers worry about the self-reported nature of qualitative data, including issues around social desirability and recall bias. We push back on this concern for qualitative data for three reasons. First, not all qualitative data is based on self-report, and analysis of photos and documents are important sources of data.^78^ Second, much quantitative research is also based on self-report and thus subject to these same biases. Third, there are some situations in which self-report can be a more accurate and meaningful depiction of a participant's experience than clinical record. This is particularly true in stress and trauma epidemiology, where participants appraisal of a situation may better represent the acuity of an exposure than an external observer.^79^ Moreover, self-reported data can be triangulated with other data sources, the concordance or divergence of which can produce important findings.

### Concern 6: data are not generalizable

Qualitative-quantitative paradigm debates have led qualitative social-constructionist researchers to reject flat-out that qualitative data is generalizable, arguing instead for “transferability” of findings depending on context.^74^ The concept of transferability places the responsibility on readers—not the research team—for deciding to what extent research findings can apply to other groups and settings.^80^ More recently, some qualitative theorists have asserted that considerations and standards for transferability in qualitative research should be the same considerations and standards for generalizability in quantitative research. That is, the context in which participants live and the research is conducted matter, and all research—regardless of whether it is qualitative or quantitative—needs to consider participant background, geography, sociopolitical environment, and historical contexts.^74^

Imbedded in this generalizability debate is a difference between probabilistic generalizability and theoretical generalizability.^26^ Emerging from the positivist tradition, probabilistic generalizability refers to the idea that a study's findings can be applied to a larger population based upon a probability sample.^26^ Theoretical generalizability instead emphasizes the contributions of a research finding to a larger theoretical understanding of a phenomenon, rather than a population,^26^ and this type of generalizability is of import to both quantitative and qualitative epidemiologists. From a social-constructionist perspective, this type of generalizability is particularly concerned with explaining variations in how individuals interact with their social environments—a key focus on social epidemiologists.^26^ Utilizing purposive sampling, in which sampling units are deliberately selected based on specific characteristics or experiences that are most relevant to the research question, can help bolster the collection of high quality qualitative data that can then be used to achieve theoretical generalizability of how a social phenomenon operates even if such findings cannot be probabilistically extended to the full population.^71^

### Concern 7: one cannot find the average

While it is true that it can be difficult to obtain measures of central tendency using qualitative data, this should not be a concern to epidemiologists. As a reminder, the CDC defines epidemiology as “the study… of the ‘distribution’*”* (emphasis added).^10^ Qualitative methods can elucidate the range of disease, disability, death, and health and their determinants and deterrents. As Daly et al. argue, the most useful qualitative studies for evidence-for-practice are the ones that capture the diversity of experiences.^81^

Take, for instance, the systematic analysis of qualitative interviews using a codebook to identify key themes or emerging issues from the data. While themes can and often do revolve around shared experiences across participants, they typically involve both breadth and depth. That is, they include both similarities and differences across the content.^82^ Parallels can be drawn to multilevel modeling, a statistical approach concerned with the context within which people and communities are nested that is popular among social epidemiologists. Multilevel modeling recognizes that while identifying the mean can be of interest, knowing the variability and heterogeneity in the population has inherent value as well.^83^ In this way, qualitative methods can advance epidemiologic goals by describing the full distribution of experiences. Take for example a study of the barriers and facilitators to managing food allergies at school. In this study, qualitative interviews highlighted that the breadth of interpersonal relationships that have an impact on students, ranging from intense instances of food allergy-related bullying to finding peer and adult champions who actively and proactively support children with food allergy.^84^

## Conclusion

The use of qualitative methods in conjuncture with epidemiologic studies is not new and has long been used in formative research and process evaluations with trials. As we argue in this paper, however, qualitative methods have value beyond such traditional uses, and if, as epidemiologists, we embrace these methods as our own, we can improve epidemiologic research at every step of the research process. This paper is not intended as “methodolotry,” and there is nothing inherently better or more progressive about qualitative methods compared to quantitative ones.^35^ Qualitative methods have strengths and limitations—as do quantitative methods—and when used appropriately in epidemiology, can strengthen public health research and translation into practice. Thus, we welcome the diverse tradition of qualitative methods into the epidemiologist's toolbox.

## Data Availability

No data were used in this paper.
